# Strainer: software for analysis of population variation in community genomic datasets

**DOI:** 10.1186/1471-2105-8-398

**Published:** 2007-10-17

**Authors:** John M Eppley, Gene W Tyson, Wayne M Getz, Jillian F Banfield

**Affiliations:** 1 Department of Bioengineering, University of California, Berkeley, CA 94720, USA; 2 Department of Environmental Science, Policy and Management, University of California, Berkeley, CA 94720, USA; 3 Department of Civil and Environmental Engineering, Massachusetts Institute of Technology, Cambridge, MA 02139, USA

## Abstract

**Background:**

Metagenomic analyses of microbial communities that are comprehensive enough to provide multiple samples of most loci in the genomes of the dominant organism types will also reveal patterns of genetic variation within natural populations. New bioinformatic tools will enable visualization and comprehensive analysis of this sequence variation and inference of recent evolutionary and ecological processes.

**Results:**

We have developed a software package for analysis and visualization of genetic variation in populations and reconstruction of strain variants from otherwise co-assembled sequences. Sequencing reads can be clustered by matching patterns of single nucleotide polymorphisms to generate predicted gene and protein variant sequences, identify conserved intergenic regulatory sequences, and determine the quantity and distribution of recombination events.

**Conclusion:**

The Strainer software, a first generation metagenomic bioinformatics tool, facilitates comprehension and analysis of heterogeneity intrinsic in natural communities. The program reveals the degree of clustering among closely related sequence variants and provides a rapid means to generate gene and protein sequences for functional, ecological, and evolutionary analyses.

## Background

With increased computational power and refinements in methods for 'shotgun' sequencing, researchers are eschewing clonal cultures in favor of sequencing microbial genomes directly from environmental samples [1-4]. This approach has the potential to revolutionize microbiology by moving beyond cultivation-based studies. Emerging techniques enable analyses of genes from uncultivated microorganisms [5-7] and genomic studies of the diversity inherent in natural populations.

The term "metagenomics" has been used broadly to encompass research ranging from cloning environmental DNA for functional screening and drug discovery [8,9] to random sampling of genes from a small subset of organisms present in an environment [3]. Some metagenomic studies aim to reconstruct the majority of genomes of the dominant organisms in microbial communities ("community genomics"). Due to current sequencing costs, near complete genome reconstruction is only possible for the dominant members of communities with a small number of organism types (e.g., AMD communities, [1]) and for a few highly abundant organisms from diverse communities (e.g., wastewater [10]). However, it is inevitable that deep sampling of additional consortia will be achieved in the near future as new sequencing technologies are deployed [11] and the costs of conventional sequencing approaches continue to fall.

Due to the random nature of shotgun sequencing, sequence data for each organism type will be obtained in proportion to its abundance in the community. Additionally, for each organism type, the average number of sequences obtained from each locus must be high to ensure most genomic loci are sampled. If near complete genome reconstruction is desired for less abundant organisms, very deeply sampled genomic datasets are acquired for more abundant organisms. In practice, DNA is extracted from so many cells that it is unlikely that any two sequences derived from the same individual [1]. Thus, 'shotgun' community genomic analyses yield genome-wide snapshots of population heterogeneity [12].

Most existing genome assembly tools were designed for assembling data from clonal isolate populations in which every individual is recently descended from, and genetically identical to, a single parental organism. While these tools successfully reconstruct genome sequences from environmentally-derived DNA [1], additional steps are needed to resolve assembly fragmentation due to insertion or loss of genes in a subset of individuals. Furthermore, the resulting fragments are composites that may not be representative of any individual in the population and mask sequence heterogeneity information that can be used to define individual level variation and the overall population structure. Thus, it is essential to develop methods to manipulate and analyze deeply sampled community genomic datasets.

Sequence variation in community genomic datasets provides information about the dynamic nature of microbial genomes [13]. Patterns of synonymous vs. non-synonymous substitutions can be modeled to identify genes under positive selection [12]. Additionally, recombination events can be identified, evidence obtained for selective sweeps of specific loci [14], and the relative rates of recombination compared to nucleotide substitution within and between species calculated [15].

In order to understand how microorganisms function within natural communities, it is essential to go beyond static snapshots of genome sequences. Minor changes in environmental conditions can dramatically change the expression profile of any given organism. Consequently, genomic information that defines the metabolic potential of an organism is not sufficient to explain its ecosystem role. However, this information can form the basis of microarray and proteomic studies to monitor changes in gene expression and protein content in response to perturbation. In theory, raw shotgun data from environmental samples could be used to compile a library of alternative gene sequences present in the population. An expanded library of potential variant sequences would have a much higher success rate in detecting genes *in situ *and, at the same time, enable strain-level resolution in functional studies. However, reconstruction of gene variant inventories for specific organisms is a formidable task without tools to visualize and analyze sequence variation in a genomic context. This endeavor will benefit from a new generation of bioinformatics tools enabling comprehensive analyses of the genomic variation at a population level captured in these data.

Here we present Strainer, a Java-based tool developed to assist in the processing of community genomic data. It is designed to aid in the visualization and exploration of genetic variation inherent in natural populations. Strainer is equipped with algorithms to enable basic analyses of within population genetic variability, including reconstruction of gene variants and mapping of recombination structure, and has the flexibility to incorporate new algorithms as new applications emerge.

## Implementation

Strainer is built around an interactive display of community genomic data and provides a suite of automated and manual tools to explore, quantify, and visualize the patterns of variation in sampled populations. Strainer uses the BioJava [16] programming framework to read and write a number of different file formats including FASTA, BLAST output, and GenBank.

### Data Preparation

Strainer displays sequence reads relative to a user defined reference sequence. The reference can be a fully assembled chromosome or genome, a contig from an assembler such as Phrap [17,18], or the genome of a related organism. Reference sequences can be input as either FASTA [19] or GenBank [20] formatted files. The latter format allows for gene annotations to be included.

Read alignments to the reference sequence can be imported into the strainer XML format from two sources. First, a contig and all aligned reads can be read directly from an ACE file produced by Phrap. Alternatively, the blastn procedural query in BLAST can be used to align reads to the reference sequence [21].

**Figure 1:**
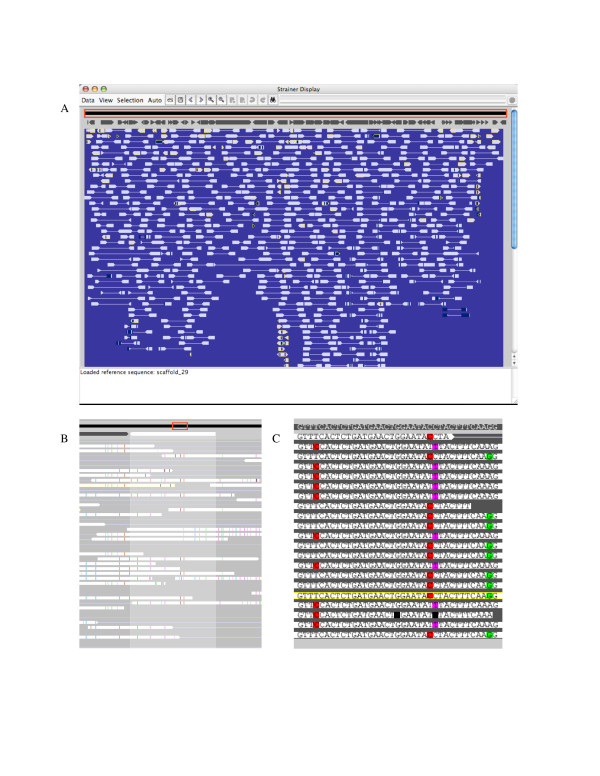
**Strainer display**. Screen captures of the Strainer program displaying scaffold 29 and read sequences from *Ferroplasma *type II community data taken from an acid mine drainage community [1]. The image in (A) shows the entire scaffold. Read alignments were determined using BLAST and are indicated with filled light-grey bars and connected to mate pairs by a thin light-grey line. Dark regions within a read indicate where the local divergence from the reference is more than 8%. The black bar at the top surrounded by a red rectangle represents the entire reference sequence (scaffold 29 in this case). The dark grey arrows immediately below indicate gene locations. Reads outlined in yellow have alignments, along with their mate pairs (not visible in this image), that are inconsistent with the size of clones. Clusters of such reads indicate an inconsistency in gene order, usually associated with transposable elements or the insertion and deleting of genes. The image in (B) shows a zoomed-in view of the same data. The red rectangle over the reference sequence bar has shrunk to indicate the location of the zoomed view. The user has selected, via a mouse click, one gene. This gene is colored white with a grey region below to indicate its extent. Reads are now white with colored tick marks where they differ in nucleotide sequence from the reference. As detailed in (C), blue, red, purple, and green ticks indicate substitutions for the bases A, C, T and G respectively. Ticks of half height indicate extra bases in a read sequence, and missing bases are colored black. Light grey ticks indicate low quality differences between the read and reference sequences.

When working with BLAST alignments, reads may align to multiple places on a reference sequence. To validate read placements, alignments are compared to corresponding mate-pair alignments. Mate-pairs are left and right end reads of a cloned DNA fragment. Reads are typically at least 700 base pairs (bp) in length, completely sequenced fragments will be obtained only from very small insert clone libraries. The size of the unsequenced region in typical libraries is not precisely known, but is constrained by the average clone size (3,000 to 5,000 bp for small insert libraries and ~40 kb for fosmid libraries). Strainer allows flexibility in mate pair placement because gene insertions in a subset of strains result in larger than expected mate pair separation in strains lacking the insert. Strainer finds alignments that place paired reads within a window of separation supplied by the user. If no such alignments are found for a pair of reads, the best individual alignments are then chosen. Yellow outlines are applied to reads that could not be placed within the user specified range of their mate-pair (the range of average clone sizes). As a result, regions in which gene order is not constant across the population are marked by yellow reads (Figure 1A).

### Visualization

Figure 1A shows an image of the Strainer interface. The black bar along the top of the window represents the reference sequence and the overlaid red frame indicates the scope of the current field of view. If a GenBank file is used, the genes are displayed as dark grey arrows immediately below this region. Bars linked with thin horizontal lines, representing aligned mate-paired reads, are displayed below. Pointed tips on bars indicate the sequencing direction and thus point to the expected placement of the paired read.

Using the toolbar, the user can zoom in or out and pan left or right to explore the read alignments. When the zoom level is high enough (when a single base is at least as wide as a pixel), colored ticks appear on each read bar to indicate, base-by-base, where the read sequence differs from the reference sequence (Figure 1B,C). By default, reads are sorted by length or by identity with the reference sequence, but can be arrayed based on read length. Clicking on the gene symbol reveals the gene position relative to the aligned reads and displays gene information in the box at the bottom of the read display.

### Coloring

There are three options for coloring read bars. The default option is to color regions within the reads to indicate locations where there are disagreements with the reference sequence. Each column of pixels is assigned one of two user-defined colors based on the percent sequence identity for a small window of bases centered at the corresponding position on the read. Both the window size for averaging and the threshold at which coloring occurs are user-defined. Regions without sequence information – e.g., the line between reads in a mate pair – are colored according to the overall sequence identity between the read and the reference. Alternatively, a single read can be shaded to an extent that depends on the percent sequence identity between that read and the reference sequence. Finally, a single solid color can be used for all reads, regardless of its identity with the reference sequence.

### Quality Data

Strainer can read in confidence values assigned to base calls (e.g., Phred scores) from a FASTA formatted quality file generated by Phred/Phrap. Base call differences with scores below a user-defined confidence level will be grayed. The user can then set a threshold confidence level below which base call differences will be deemphasized (colored gray). Strainer allows the user to specify whether unknown bases (N's or low quality bases) should be ignored when calculating sequence divergences.

### Read Groupings and Apparent Recombinant Reads

A major goal is to group reads with similar sequences so as to reconstruct variant gene sequences. In the manual strain reconstruction mode, the user clicks on reads to select them (selected reads are highlighted in blue) and then uses the "Make Strain" button to bring all the selected reads into a single strain fragment indicated by a surrounding colored rectangle. Similarly, strain groups can be joined. Strain fragments are given random colors by default, or can be colored using the same 3 methods available for reads. This choice is independent of the read coloring method chosen. Grouping into strain fragments will often highlight reads that are divergent over only a portion of their length. This may be due to insertion of sequence (e.g., a transposon in only a subset of individuals) or to homologous recombination with another sequence type. Recombinants can be recognized most easily as chimeras of two variant sequences. The user can flag such reads as recombinants (the program will outline them in red) and assign the mate pairs to the most relevant strains.

### Automatic Generation of Read Groups

**Figure 2:**
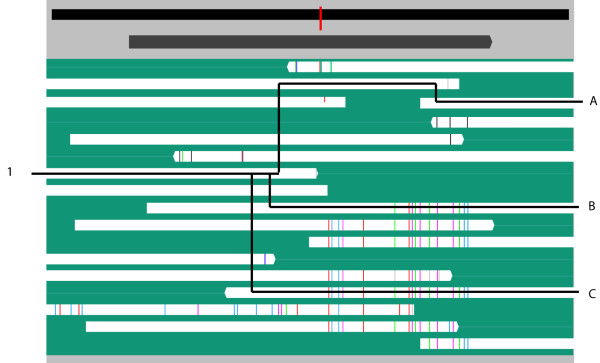
**Building variant sequences**. Illustration of the variant enumeration algorithm. Data taken from Tyson *et al*. 2004 [1] for *Ferroplasma *type I. Starting with the read labeled (1), three different paths are shown (A, B, and C) that span the gene by linking reads which overlap with no differences. To determine the sequence variants present in the data, all paths are found using all possible starting reads.

Strainer includes an algorithm to reconstruct all possible variant sequences by considering all legitimate linkage pathways. Reads with overlapping sequence patterns are linked to form more extended strain sequence types so long as overlap exceeds a user-specified threshold. In cases where alternative variant paths are possible, such as when overlapping sequence subsequently diverges into two or more paths (Fig. 2), both potential paths are generated. The algorithm can be executed on any chosen gene, for every gene in the annotation, or for any user defined segment of the genome. The variants can be output as a FASTA list of nucleotide or amino acid sequences.

Strainer can also use this algorithm to automatically group reads in the display into strain types. Since variants are determined by exploring all the possible ways to link reads, a single read can be associated with multiple variant sequences. In instances where reads can be assigned to multiple groups, reads are placed into the largest group. Groups generated automatically can be manually curated to resolve complicated regions, such as conflicting read placements due to recombination.

### Group Sequences and Read Lists

Strainer allows the user to select a series of reads or strain fragments and export the composite sequence to a FASTA file as either nucleotides or amino acids. Regions containing gaps in sequence coverage can either be filled in from the reference sequence or marked with N's (or X's for amino acid sequences). In addition, nucleotide or amino acid sequences of all strain variant groups for all genes can be output. Also, a list of reads contained in strain fragments can be output to a text file for use in other applications.

### Editing the Consensus Sequence

Assembly of closely related sequences can generate a composite sequence that is a mosaic of strain types and is not actually found in the environment. The composite may also take on the sequence of a less abundant variant. Therefore, it is important to have the ability to alter the composite sequence after strain analysis. Strainer can alter the reference sequence to match a selected strain, read, or single base pair. The updated reference sequence can be exported to a FASTA file for ORF searches or other applications.

### Obtaining Strainer

Strainer was developed in Java to enable seamless execution in almost any computing environment. It is available for download as a self-contained application (see below). Additionally, the source code and more detailed documentation are available online. A programming interface (API) is provided and described in the online documentation (see below) to allow custom algorithms (such as new clustering methods) to be implemented, if desired.

## Discussion

Strainer was specifically developed to aid in analysis of community genomic data recovered from the Richmond Mine acid mine drainage (AMD) community [1,15,22]. Consequently, it was designed to facilitate the exploration and analysis of variability within and between closely related populations sampled by comprehensive genomic datasets. The program is most useful for sampled populations where, on average, each base in the genome is represented in at least 3 reads (~3X coverage), as overlapping sequences are required to see within-population structure. It will be applicable to new high coverage datasets obtained from other environments. The interface was constructed to suit data from small insert libraries but also could be used for analysis of shorter sequences generated by new sequencing technologies such as GS20 pyrosequencing (Roche, Inc).

In this paper, we demonstrate Strainer by applying it to analyze variability in genomic data from the AMD system. The figures illustrate analyses of several community members using different modes of Strainer imports. In some cases, reads were aligned by blastn to either an isolate genome or a composite scaffold generated by assembly of metagenomic data. In other cases, reads assembled into contigs by Phrap were manually curated in Consed and the contig and aligned reads imported into strainer. Thus, Strainer is applicable to a variety of different input data types. Base calling quality data (Phred scores) were imported when available to indicate differences that are likely due to sequencing error.

**Figure 3:**
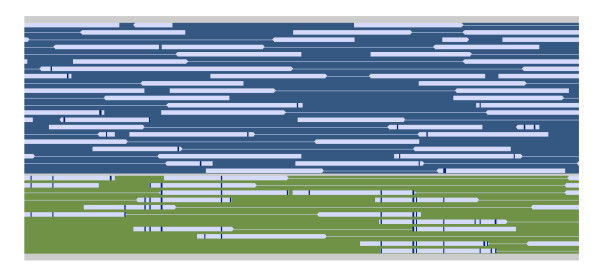
**Clusters in *Leptospirillum *group II**. Reads in *Leptospirillum *group II are grouped into two distinct types. Here reads are colored using the two-toned approach. Dark vertical bars within reads indicate regions that contain SNPs. Colored backgrounds (blue and green) indicate strain designations.

Strainer can assist in binning of DNA sequence fragments to organism types. When binning community genomic data, reads can be aligned to an isolate genome sequence using blastn if they exceed an identity threshold (as was done in a binning step by Tyson et al. [1] and more recently by Yooseph et al [23]). This is most effective if there is significant genome synteny. Alternatively, as was done here, reads can be aligned simultaneously to one or more isolate genomes and all reconstructed genome fragments so as to find the best placement of each read prior to refinement of bins. For example, in Figures 1 and 2 reads were assigned to *Ferroplasma *type I if they aligned to the *F. acidarmanus *reference sequence with an e-value less than 1 × 10^-25 ^and had no better alignment to other community genome fragments. Analysis of patterns of sequence variation among reads using Strainer can reveal clear sequence clusters. This is illustrated in Figure 3. In cases such as this, binning could be refined through stringent assembly or by strain variant reconstruction using the Strainer read grouping tools.

**Figure 4:**
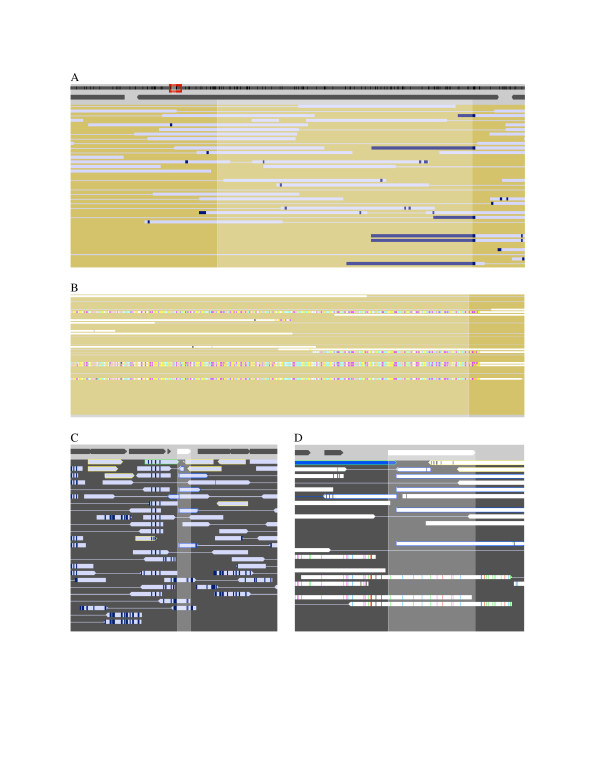
**Gene order differences**. Illustrations of gene order differences appearing in Strainer. Reads from *Leptospirillum *group II were assembled into contigs by Phred/Phrap. One contig and its component reads were imported from the Phrap generated ACE file and are shown in (A). Reads with gene orders that do not match the reference sequence stand out due to high levels of differences. Panel (B) shows a segment of the same region at higher zoom revealing similar patterns in all the differing reads. In (C), reads from *Ferroplasma *type I were aligned against the *Ferroplasma acidarmanus *isolate genome using blastn. BLAST cuts off alignments when the similarity ends, but gene order differences are still apparent due to multiple reads being clipped (in blue) at the same point and a cluster of reads (in yellow) missing mate-pairs. Panel (D) shows the same region at higher zoom revealing the exact point at which the read alignments are trimmed.

Strainer-based analysis may assist in identification of assembly errors, such as those that arise due to repetitive sequences. Although these can be identified in other ways, the ability to rapidly survey large genomic regions for mate pair inconsistencies reduces the effort needed to screen very large community genomic datasets. More importantly, the assembled reads can be scanned for evidence of gene insertion in a subset of individuals. Such areas may be evident due either to unusually long (or short) mate-pair placements or inconsistencies with the reference sequence (Figure 4). If data are imported from assemblies, reads with gene insertions or deletions may have markedly different sequence on one end (<50% identical; Figure 4A). Such features are apparent, even at low zoom levels. When reads are imported from BLAST alignments, gene gain or loss in more than one read will be marked by coincident truncation of alignments (Figure 4B).

The strength of Strainer is its ability to convey the nucleotide variability within a population. Because the program can compare reads from one population to the composite or isolate sequence(s) from related organisms, it can be used to identify conserved non-coding regions that may be involved in gene regulation. It can also be used to visualize patterns of distribution of nucleotide substitutions within genes and provide information about patterns of evolutionary relatedness amongst strains.

**Figure 5:**
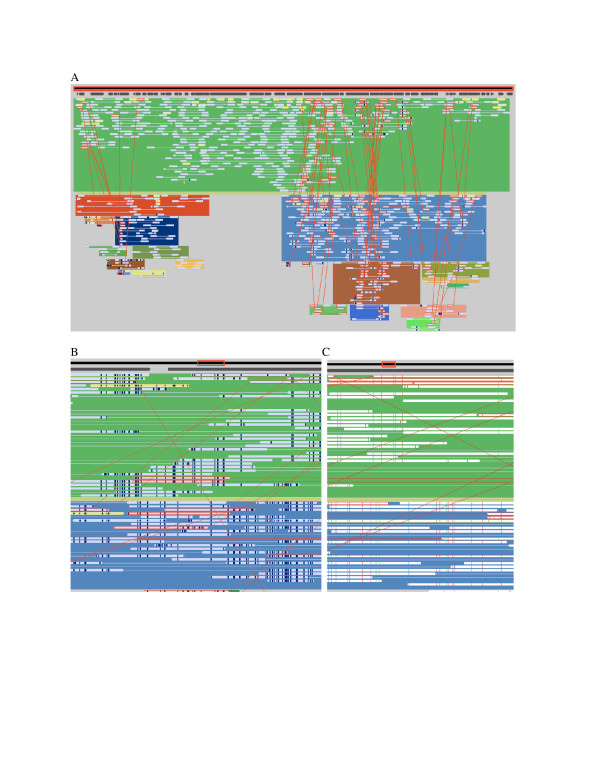
**Grouping reads and labeling recombinants**. A "strained" scaffold shown at different zoom levels: (A) full scaffold, (B) a few thousand bases, (C) viewing individual SNPs. Environmental reads were aligned to scaffold 29 from *Ferroplasma *type II and manually grouped into strains. Reads placed in different strains from their mate-pairs are marked with red outlines and are linked to their mate-pairs by diagonal red lines. Also marked in red are read pairs which are placed in the same strain, but at least one read had a small region that matched a different strain.

Inconsistencies in groupings based on single nucleotide substitutions are amongst the most interesting patterns that can be readily surveyed using Strainer. Linkage between different variant types may be indicative of recombination. Even if strain fragments cannot be definitively extended over large genomic regions, it is possible to analyze recombination locally. Parent and recombinant strain types can be assigned by using a configuration that minimizes the number of recombinant compared to non-recombinant (parent) reads. Using the manual interface, it is possible to link fragments together into the chosen configurations, highlighting apparent recombinant reads in the process (Figure 5). Thus, Strainer permits rapid analysis of the distribution of recombination events across genomes. In addition, the program facilitates quantification of the relationship between incidence of recombination and nucleotide divergence between the parent sequence types, as described by Eppley, Tyson et al. [15].

A central capability of Strainer is its ability to facilitate reconstruction of gene variant sequences from population (i.e., from sequences from individuals that are closely related enough that their sequences coassemble) genomic datasets. These sequences can be compared to each other and to other database sequences for analyses of distributions and ratios of synonymous vs. non-synonymous (dN/dS) nucleotide substitutions. Thus, Strainer provides a route for genome wide identification of genes under selection.

One of the most exciting opportunities presented by deeply sampled community genomic datasets is the ability to analyze the functions of coexisting microorganisms in their natural environments. For methods such as microarray-based gene expression studies and mass spectrometry-based proteomics [24], comprehensive genomic datasets are key. However, the resolution of these methods will depend on the accuracy of the predicted gene and proteomic sequences. For example, it is possible to use mass spectrometry approaches to identify proteins from species reasonably closely related to the organisms of interest [7,22]. However, peptides that differ from predicted peptides will generally not be identified, especially in high throughput (shotgun) proteomics experiments. This motivates the development of catalogues of possible protein variants that can be used to increase the resolution of functional analyses [22]. For example, of the 1,973 genes identified in the *Ferroplasma *type 1 isolate genome, 1,881 genes are at least partially covered by reads from the environmental sample. For each of these, Strainer produced a list of possible variant sequences. On average, Strainer found 6 possible variant nucleotide sequences for each gene (average divergence 1.9%) corresponding to an average of 5 amino acid variants for each gene (average divergence 2.1%).

Strainer is a first generation metagenomics tool that has been specifically designed so that its capabilities can be expanded to meet new needs and opportunities. Future expansions could include display of synonymous vs. non-synonymous substitutions and gene-by-gene calculation of dN/dS values. We envision integrating functional information into strainer so as to enable direct comparison of the activity of closely related organisms within a single community. In addition, the platform could be extended to create a tool for more complete rendering of population genomic data, capturing information from sequence fragments that were separately assembled due to heterogeneities in gene content. Its flexibility allows for the integration of new algorithms for sequence clustering and improved methods for read alignment. Rather than exhaustively develop the Strainer program to include all such features, we are releasing the software as open source so as to engage the broader community in its testing and development.

## Conclusion

The software presented in this paper enables researchers to gather and explore information from deeply sampled metagenomic (community genomic) datasets and to analyze genetic variability that is masked by the composite sequence. This information provides valuable insight into population structure and evolutionary dynamics, and greatly enhances the effectiveness of functional studies. Strainer was built in Java to be completely platform independent and uses the BioJava framework to share data easily with other bioinformatics tools.

## Availability and Requirements

Strainer and its source code are available freely under the terms of the Lesser Gnu Public License. It is hosted as the "Strainer" project at Bioinformatics.org: . Strainer is platform independent and will run on any system with a Java 1.5.0 or later runtime environment. Input files are expected to be in BLAST, GenBank, Ace, or FASTA formats. Phrap or BLAST need to be obtained separately for generating these files.

## List of abbreviations used

• BLAST – Basic Local Alignment Search Tool: A rapid sequence matching tool. (blastn is the nucleotide specific algorithm within BLAST).

• Contig – A contiguous segment of sequence assembled from overlapping sequence reads. One or more contigs are linked by mate-pair associations into scaffolds.

• FASTA – FAST All: A sequence alignment method and program (Pearson and Lipman 1988) whose file format has become the standard for simple sequence data.

• GenBank – Genome Bank: A public repository of sequence data maintained by NCBI whose file format has become a standard for sequence data with annotations.

• NCBI – National Center for Biotechnology Information

• Phrap – Phragment Assembly Program: A freely available program for assembling shotgun sequencing reads into contigs.

• Phred – A freely available program for generating nucleotide sequences from raw instrument data.

• SNP – Single Nucleotide Polymorphism: in a sequence alignment, a nucleotide position at which sequences disagree.

## Competing interests

The author(s) declares that there are no competing interests.

## Authors' contributions

JME coded the software, assisted in software design, performed the analyses for this article, and drafted the manuscript. GWT assisted in software design and testing and obtained and prepared data. WMG contributed to design of the project and preparation of the manuscript. JFB initiated the genomic sequencing component of the project, assisted in software design and testing, and in preparation of the manuscript. All authors read, edited, and approved the final manuscript.
